# Effect of Cognitive Stimulation Therapy on Cognition and Social Independence in People With Dementia at the North Norfolk Memory Service

**DOI:** 10.1192/bjo.2024.96

**Published:** 2024-08-01

**Authors:** Oluwaseun Olaluwoye, Gemma Ridel, Bethan Jones, Abosede Ighomereho

**Affiliations:** Norfolk and Suffolk NHS Foundation Trust, Norwich, United Kingdom

## Abstract

**Aims:**

Cognitive stimulation therapy (CST) is a psychosocial treatment for people with dementia. It is an evidence-based treatment which shows improvement in cognition, well-being and quality of life of people living with dementia. CST is recognised as one of the interventions which is cost-effective.

The National Institute of Health and Care Excellence (NICE) guideline recommends that people with mild to moderate dementia should be given opportunities to take part in CST. The CST sessions done in North Norfolk are weekly sessions which span 90 minutes. A group of between 8–10 people attend a 14-week CST treatment course.

The aim of this study is to evaluate the effectiveness of the CST on cognition and social independence of patients with dementia in North Norfolk.

**Methods:**

Patients diagnosed with mild to moderate dementia at the memory service were referred for CST sessions. A trained professional assessed the patients to determine their eligibility for CST. The assessment included: assessing motivation to join a group therapy, administration of the short-version of the Addenbrooke Cognitive Examination questionnaire (MINI-ACE) to assess cognitive functions and the administration of Engagement and Independence in Dementia Questionnaire (EIDQ) which measures the social independence of the patients. A higher score on both questionnaires indicates better cognitive function and social independence, respectively.

The CST sessions spanned from February 2023 to May 2023. The patients were re-assessed after the 14-week sessions of CST for their MINI-ACE and EIDQ scores. A qualitative questionnaire was administered for feedback about the sessions.

Data were obtained from patients' clinical record following approval from the research and service evaluation team of the Trust.

**Results:**

Nine patients completed the 14-week CST sessions. The mean age of the patients was 82.9 ± 4.8. 66.7% were male and 33.3% were female. 77.8% were on memory medication and 22.2% were not on memory medication. 44.4%, 33.3%, 11.1% and 11.1% were diagnosed with dementia in Alzheimer's Disease; Mixed Alzheimer's-Vascular Dementia, Lewy Body Dementia and Frontotemporal Dementia, respectively.

The same proportion of patients (44.5%) had both increased and decreased MINI-ACE score after CST while 11% had no changes in MINI-ACE score. Majority (66.7%) had increased EIDQ score after CST, 22.2% had decreased EIDQ score and 11.1% had no changes.

**Conclusion:**

The CST sessions done in North Norfolk showed more positive effect on social independence than cognition in people with dementia.

